# Electrochemical copolymerization of 3,4-ethylenedioxythiophene and dithienothiophene: influence of feed ratio on electrical, optical and electrochromic properties†

**DOI:** 10.1039/d3ra08729h

**Published:** 2024-04-08

**Authors:** Rashi Kedia, Manisha Khatak, Manisha Balkhandia, Asit Patra

**Affiliations:** a Photovoltaic Metrology Section, Advanced Materials & Device Metrology Division, CSIR-National Physical Laboratory Dr K. S. Krishnan Marg New Delhi 110012 India apatra@nplindia.org; b Academy of Scientific and Innovative Research (AcSIR) Ghaziabad 201002 India

## Abstract

Designing a copolymer is an efficient and alternative method to generate new chemical and physical properties compared to parent homopolymers without complex synthesis and structural modification. We herein report the electrochemical deposition of copolymer using two monomers 3,4-ethylenedioxythiophene (EDOT) and dithieno[3,2-*b*:2′,3′-*d*]thiophene (DTT). Three different copolymers P[EDOT-*co*-DTT] were synthesized by using different feed ratios of monomers (EDOT and DTT molar ratios in solution are 2 : 1, 1 : 1 and 1 : 2) in acetonitrile containing 0.1 M tetrabutylammonium perchlorate (TBAClO_4_) as a supporting electrolyte. Fourier transform infrared spectroscopy (FTIR), Raman spectroscopy and UV-vis-NIR spectroscopy were employed to characterize the obtained copolymers. Energy dispersive X-ray spectroscopy (EDX) analysis was used to estimate the composition of EDOT and DTT units in copolymers. The electrochemical and morphological properties were analyzed using cyclic voltammetry (CV) and field emission scanning electron microscopy (FESEM). *In situ* spectroelectrochemistry and electrochromic studies were performed to investigate the optical and switching properties of the resultant copolymers. The homopolymers poly(3,4-ethylenedioxythiophene) (PEDOT) and polydithieno[3,2-*b*:2′,3′-*d*]thiophene (PDTT) were also prepared using similar electrochemical conditions and made comparisons where applicable. Computational calculations were done to understand the structure and energy levels of these polymers. It was found that these copolymers P[EDOT-*co*-DTT] show new properties as compared to homopolymers PEDOT and PDTT for organic electronic applications. Interesting to note that the resultant copolymers display the property of tunable electrochromism with improved transmittance and redox color change between the neutral and oxidized states.

## Abstract 2

Three different copolymers P[EDOT-*co*-DTT] were electrochemically synthesized by using different feed ratios of monomers in MeCN/TBAClO_4_ medium. These copolymers show new properties as compared to homopolymers for electronic applications.

## Graphical abstract

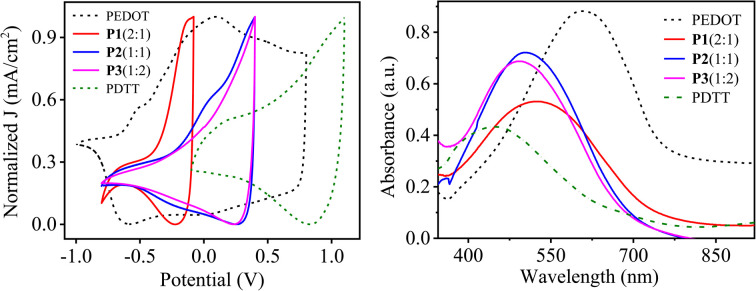

## 1. Introduction

Conjugated polymers have drawn significant attention in the field of optoelectronics applications due to their tunable properties and versatile fabrication approach.^1–4^ They have been widely used in organic solar cells, energy storage, chemical sensors, electrochromic devices, organic light-emitting diodes, actuators, field effect transistors and so forth.^5–11^ Synthesis of new monomer units is a common approach for the modification of chemical and physical properties of conjugated polymers such as solubility, stability, optical and electrical, *etc.*, but it requires multi-step synthesis, time-consuming and costly process. Alternatively, various types of conjugated polymers, such as copolymer, donor–acceptor, block, end-group functional polymers, *etc.*, have been designed and synthesized to achieve desirable chemical and physical properties for organic electronic applications.^12,13^ Among them, copolymerization of two distinct monomer units is a simple method to generate new properties compared to parent homopolymers without complex synthesis.^14,15^ Considerable efforts have been made to synthesize copolymers in which two or more suitably functionalized monomers are copolymerized together in search of intensified properties to those of the individual homopolymers.^16–20^

Substituted polythiophenes, especially 3,4-ethylenedioxythiophene (EDOT) based polymer, has been of particular research interest because of captivating properties of PEDOT such as high conductivity, superior environmental stability, low oxidation potential, fast switching between conducting doped and insulating undoped form, high transparency in the oxidized state.^21^ However, the low band gap of PEDOT allows the color change from dark blue to light blue only upon oxidation which confines the application of homopolymer PEDOT in some cases.^22^ Further, the higher-lying HOMO energy level of PEDOT leads to oxidization under ambient conditions. Precisely for PEDOT-based copolymers, Yi-Jie *et al.* showed the copolymerization of EDOT with pyrrole, which displays a wide range of color variation from purple-red, brick-red, and dark-grey to light blue.^23^ Camurlu and coworkers reported the copolymers between EDOT and 1-(perfluorophenyl)-2,5-di(thiophen-2-yl)-1*H*-pyrrole (FPTPy) to achieve desired electrochromic properties with multiple color variations and good switching times.^24^ Hu *et al.* reported the electrochemical synthesis of copolymer from EDOT and perylene units, which showed four color changes from reddish brown to light green at the different doped states.^25^ Feng *et al.* electrosynthesized multielectrochromic copolymer of 1-(3-methylthiophen-2-yl) pyrene and EDOT to fabricate electrochromic device based on copolymers which showed better properties than corresponding homopolymers.^26^ The copolymerization between EDOT and pyrene resulted in good thermal stability and smooth morphology.^27^

On the other hand, polydithieno[3,2-*b*:2′,3′-*d*]thiophene (PDTT) has fused aromatic rings, which may lead to a better conjugation in the polymeric backbone for intermolecular charge transfer.^28^ Polymer PDTT shows a band gap at 1.96 eV with p-doping behaviour and exhibits visible colour change between the undoped and doped forms.^29–31^ The colours of the neutral and oxidized PDTT film are red-orange and blue-grey, respectively. However, the electrochromic behaviour of PDTT film is difficult to be exploited mainly due to the degradation of the film on repetitive colour switching due to the instability of the oxidized state.^32^

Generally, the properties of copolymers mainly depend on the nature of precursors, along with the minor effect of polymerization technique and reaction conditions. In this connection, it is expected that electro-copolymerization between EDOT and DTT might produce new and improved properties, which could overcome the limitation of corresponding homopolymers. Electrochemical synthesis is an efficient one-step approach to produce copolymer films.^33^ The advantages of electrochemical deposition are (i) small amounts of precursor are required and high purity polymer films are obtained (ii) the technique is fast and environmentally friendly (iii) electrochemical reactions are carried out at room temperature, (iv) controllable parameters such as the thickness of film, deposition time, *etc.*^34–36^ Moreover, an effective electrochemical copolymerization occurs when the oxidation potentials of two monomers are close to each other.^37,38^ Herein, both monomers EDOT (oxidation potential 1.11 V) and DTT (oxidation potential 1.07 V) could be oxidized simultaneously within the same potential range and react with each other to form copolymers.

In continuation of our work on the conjugated copolymers,^39–44^ three copolymers based on PEDOT and PDTT were synthesized by using different feed ratios of monomers EDOT and DTT as 2 : 1, 1 : 1 and 1 : 2, respectively. Copolymer films were electrodeposited on the ITO-coated glass surface by electrochemical oxidation of monomers EDOT and DTT, *via* electro-copolymerization method in 0.1 M TBAClO_4_/MeCN solution. The resulting copolymer films were characterized by Fourier transform infrared (FTIR) and Raman spectroscopy. The elemental composition of copolymer films was examined by using an energy-dispersive X-ray (EDX) spectrophotometer. The electrochemical and morphological properties were analyzed using cyclic voltammetry (CV) and field emission scanning electron microscopy (FESEM). The optoelectronic properties of the prepared copolymer films were examined *via* spectroelectrochemistry in 0.1 M TBAClO_4_/MeCN solution. Computational studies were carried out to understand the theoretical optimized geometry, HOMO, LUMO and band gap of the copolymer P[EDOT-*co*-DTT]. Finally, the electrochromic properties of the obtained copolymer films were examined, and the result shows the property of tunable electrochromism with improved transmittance and redox color change between the neutral and oxidized states.

## 2. Experimental section

### 2.1 Materials

3,4-Ethylenedioxythiophene (EDOT, 97%), dithieno[3,2-*b*:2′,3′-*d*]thiophene (DTT, 97%) and tetrabutylammonium perchlorate (TBAClO_4_, for electrochemical analysis ≥99.0%) were purchased from Sigma-Aldrich. Both EDOT and DTT were used as received while TBAClO_4_ was oven-dried at 60 °C for 12 h before use. Acetonitrile (MeCN, 99%) was purchased from Finar Chemicals, which was purified over calcium chloride by refluxing and distillation methods. The working electrode indium tin oxide coated glass (ITO, *R*_s_ < 10 Ω sq^−1^) was purchased from Shilpa Enterprises, Nagpur, India and was cleaned with deionized water and acetone, respectively. Potassium bromide (KBr, 99.5%) was bought from Sisco Research Laboratories Pvt. Ltd India. Other chemicals were used as received without further purification if otherwise stated.

### 2.2 Electrochemical copolymerization

Electrochemical synthesis of copolymers based on PEDOT and PDTT was performed *via* the potentiodynamic process in a one-compartment cell equipped with three electrodes. ITO-coated glass (dimension: 7 mm × 50 mm × 1.1 mm, *R*_s_ < 10.0 Ω sq^−1^), gold (Au) wire and silver (Ag) wire were used as a working, counter and reference electrode, respectively. All three electrodes were immersed in MeCN solution containing 0.1 M TBAClO_4_ as a supporting electrolyte. The two monomers EDOT and DTT were added to the above solution in various feed ratios (by mole) *i.e.*, 2 : 1, 1 : 1 and 1 : 2, respectively, to synthesize three different copolymers P1(2 : 1), P2(1 : 1) and P3(1 : 2), individually (Table S1†). Before electro-copolymerization, the resultant solutions were deoxygenated by purging N_2_ gas for 5 min. At scan rate of 100 mV s^−1^ and in the potential range of −0.8 to 1.5 V, the corresponding copolymers P1(2 : 1), P2(1 : 1) and P3(1 : 2), respectively, were electrodeposited on the surface of ITO-coated glass substrate. The obtained copolymer films were rinsed in MeCN to remove unreacted monomers and residual electrolytes. For comparison purposes, homopolymers PEDOT and PDTT were prepared by electrochemical polymerization of monomer EDOT and DTT respectively under similar conditions as described above for copolymerization.

### 2.3 Equipments and characterizations

Electrochemical copolymerization and the electrochemical measurements were carried out by cyclic voltammetry on Metrohm Autolab, PGSTAT204 potentiostat equipped with NOVA software. A conventional single-compartment, three-electrode system was employed for electrochemical setup with Ag wire as reference, Au wire as counter and ITO-coated glass slide as working electrode. The reference electrode was calibrated externally by adding 0.001 M solution of ferrocene in a degassed electrolytic solution of 0.1 M TBAClO_4_/MeCN. The oxidation peak of the ferrocene/ferrocenium (Fc/Fc^+^) redox couple was found to be 0.4 V *vs.* Ag/Ag^+^. All the electrochemical investigations were performed at the room temperature. For the structural characterization, as prepared copolymer samples were dispersed (by scratching from the ITO surface) in potassium bromide (KBr) to make pellets for FTIR analysis and then spectra were recorded on a PerkinElmer Spectrum-2 spectrophotometer in the wavenumber range of 4000 to 400 cm^−1^. The Raman spectra of the resultant copolymer films were obtained by using a T64000 triple Raman spectrometer with a 514 nm laser. The absorption spectra of the monomers solution having different feed ratios of EDOT and DTT (2 : 1, 1 : 1 and 1 : 2 by mole) were recorded before and after the electro-copolymerization process by using a UV-1800 Shimadzu spectrophotometer over the wavelength range from 200 to 1100 nm. The surface morphology of the copolymer films was analysed by field emission scanning electron microscope (FESEM, Zeiss Supra 40VP), connected to a dispersive energy X-ray (EDX) microanalyzer for the elemental analysis at an accelerating voltage of 5.0 kV. Before the characterizations, the electrodeposited copolymer films on ITO-coated glass were freely removed from the electrochemical cell and rinsed in MeCN to wipe out unreacted monomers and residual electrolytes. Further, as-prepared copolymer films were dried under N_2_ atmosphere. Prior to FESEM analysis, the dried films were mounted on copper stubs and sputter-coated with an ultrathin layer of gold to avoid charging during the measurements. *In situ* spectroelectrochemical measurements were conducted in a monomer-free electrolytic solution (0.1 M TBAClO_4_/MeCN) using a quartz cuvette by applying the different potentials. The optical band gap (*E*_g,opt_) of the copolymers were calculated from the onset absorption wavelength of the copolymer, obtained by extrapolating the linear portion of the curve to the wavelength axis (*X*-axis). Electrochromic properties of the copolymer films were investigated by using the chronoamperometry technique at *λ*_max_ in the potential range from −1.0 to 1.0 V.

## 3. Result and discussion

### 3.1 Electrochemical copolymerization

**Scheme 1:**
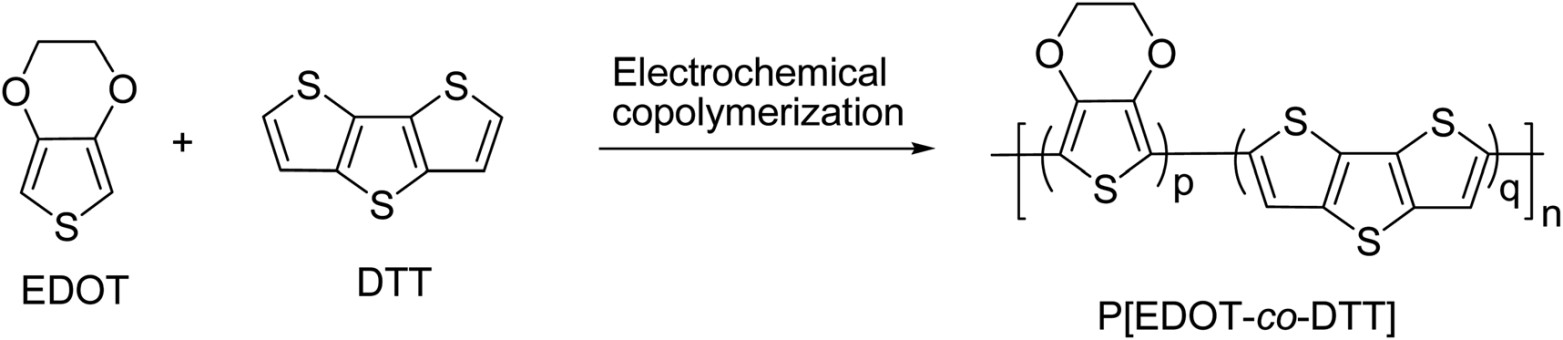
Electrochemical copolymerization of the monomers EDOT and DTT to obtain copolymer P[EDOT-*co*-DTT]. Three different feed ratios (molar ratio) of EDOT and DTT 2 : 1, 1 : 1 and 1 : 2 was used to prepare copolymers P1(2 : 1), P2(1 : 1) and P3(1 : 2), respectively in 0.1 M TBAClO_4_/MeCN solution.

**Fig. 1:**
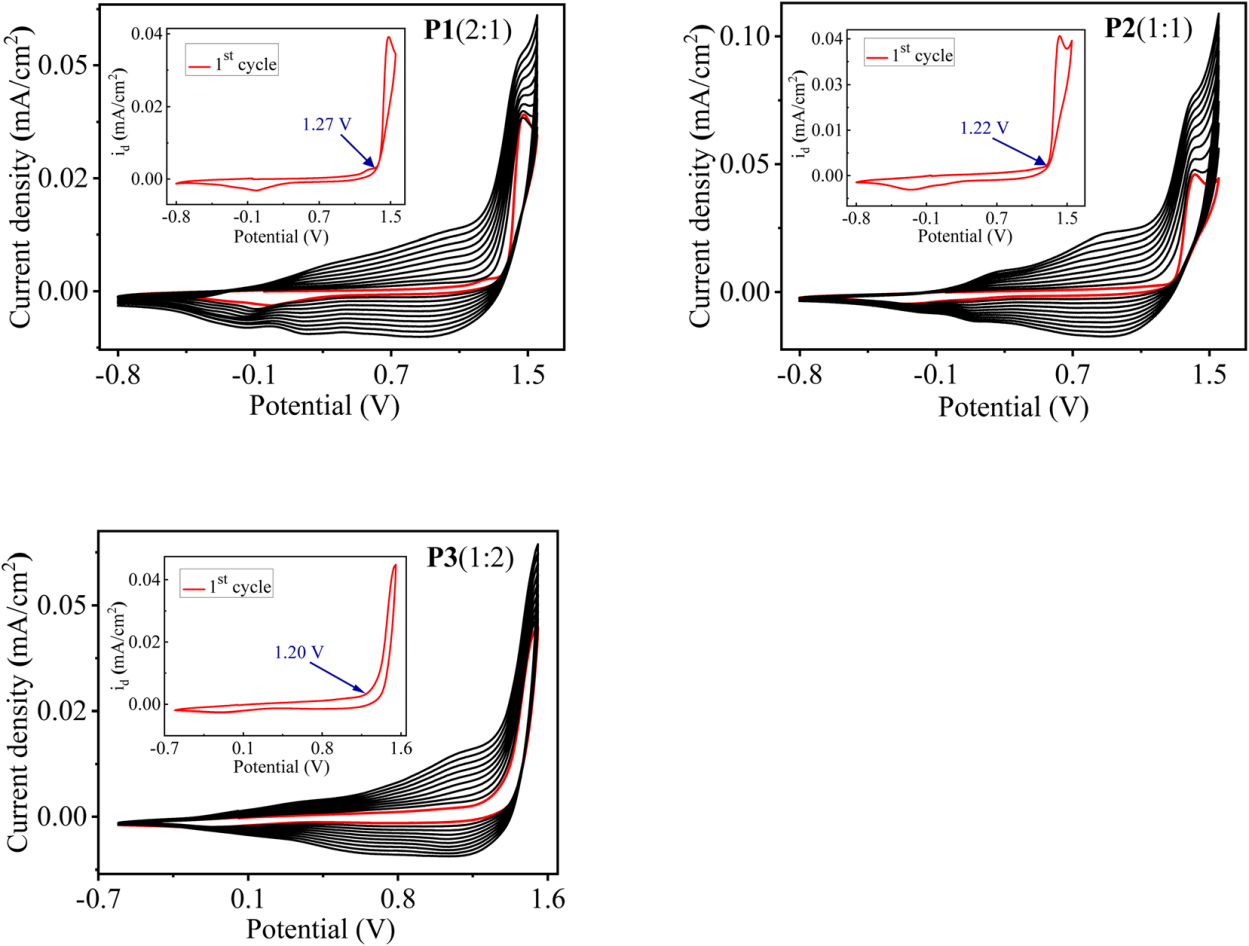
Electrocopolymerization curves of the monomers EDOT and DTT with different feed ratios as 2 : 1, 1 : 1 and 1 : 2 in 0.1 M TBAClO_4_/MeCN solution to electrodeposit copolymers P1(2 : 1), P2(1 : 1) and P3(1 : 2), respectively at scan rate of 100 mV s^−1^ (*vs.* Ag/Ag^+^ wire, Fc/Fc^+^ = 0.4 V). The inset shows the first oxidative scan of the monomers.

The electrochemical synthesis route of copolymer P[EDOT-*co*-DTT] is illustrated in Scheme 1. Three different feed ratios of monomers (molar ratio of EDOT and DTT) *i.e.*, 2 : 1, 1 : 1 and 1 : 2 in 0.1 M TBAClO_4_/MeCN were used to obtain three different copolymers films on the ITO-coated glass slide and are denoted as P1(2 : 1), P2(1 : 1) and P3(1 : 2), respectively. The electrochemical copolymerization curves of monomers with various feed ratios as 2 : 1, 1 : 1 and 1 : 2 is shown in Fig. 1. The first cycle corresponds to the irreversible oxidation of monomers and is also depicted in the inset graph of Fig. 1. The onset oxidation potential (*E*_onset_) of monomers was found to be 1.27, 1.22 and 1.20 V, corresponding to three different feed ratios of monomers as 2 : 1, 1 : 1 and 1 : 2, respectively. It is worth noticing that *E*_onset_ of monomers is higher than those of individual monomer EDOT (1.11 V) and DTT (1.07 V), indicating the existence of interaction between the monomers in 0.1 M TBAClO_4_/MeCN solution. *E*_onset_ of the individual monomer EDOT and DTT were extracted from their electropolymerization curves, as illustrated in Fig. S1.† It is well-known that a successful electrochemical copolymerization occurs when the *E*_onset_ of different monomers are close to each other.^22,35^ In our case, it was found that the difference in the *E*_onset_ of the EDOT (1.11 V) and the DTT (1.07 V) is as low as 0.04 V, which suggests the feasibility of electro-copolymerization. From this point, the monomers EDOT and DTT are revealed to get oxidized within the same potential range and radical cations of both monomers might form simultaneously on the ITO surface where they can react with each other and form a copolymer.

As shown in Fig. 1, from the second cycle onwards, a new reversible redox couple appears, indicating the deposition of polymer on the ITO electrode and denotes the redox behaviour of the electrodeposited polymers. The current density increases with the increasing number of cycles, which shows the gradual deposition of well-adhered, conducting, insoluble polymer film on the ITO surface and suggests an effective copolymerization process. Moreover, the increment between consecutive cycles and redox behaviour of the copolymers is completely different from those of their parent homopolymers PEDOT and PDTT, which confirms the formation of polymers consisting of both EDOT and DTT units.

### 3.2 Absorption spectra of monomers before and after polymerization

**Fig. 2:**
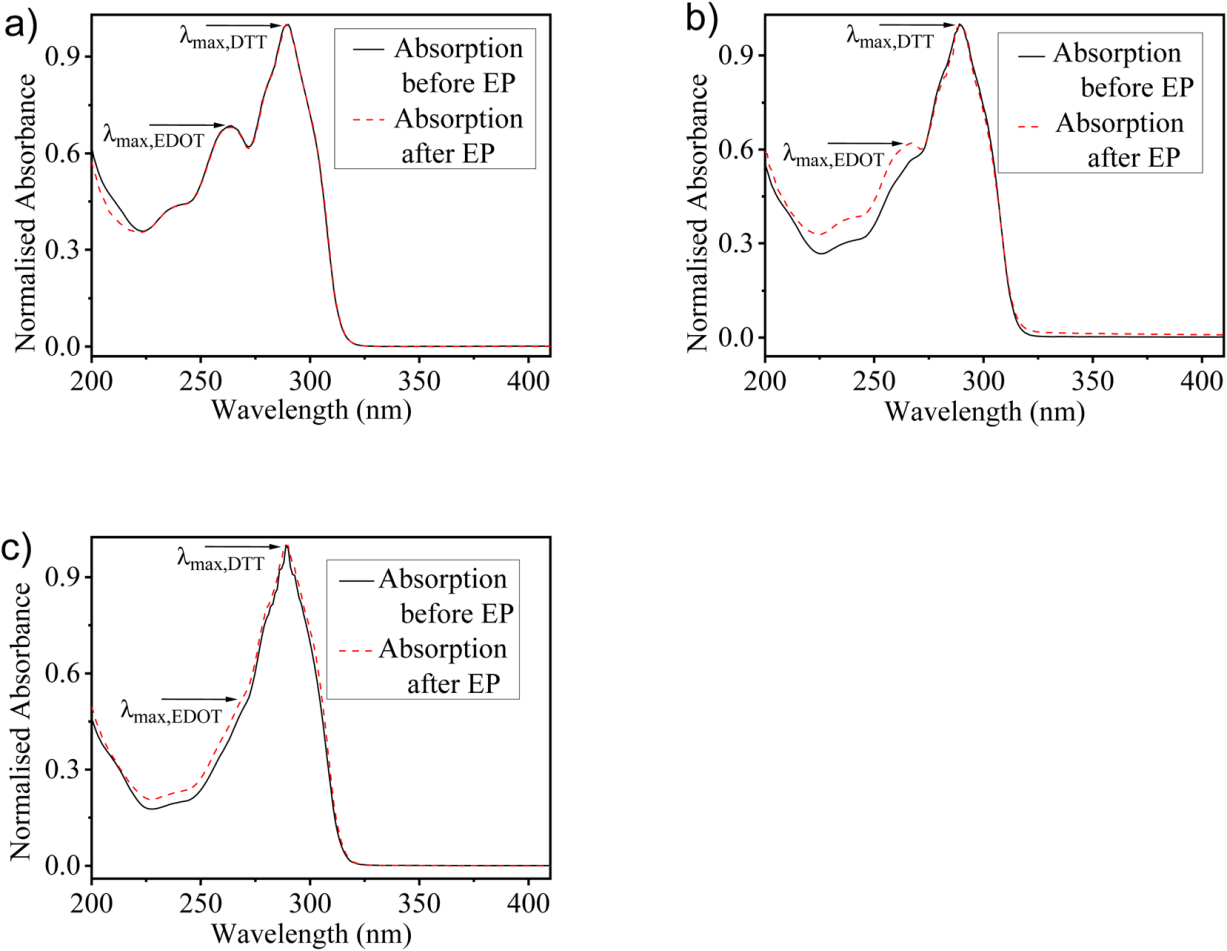
UV-visible absorption spectra of the mixture of monomers in MeCN solution before and after the electro-copolymerization process, prepared with the different feed ratios of EDOT : DTT as (a) 2 : 1, (b) 1 : 1 and (c) 1 : 2. (EP = electro-copolymerization).

The optical spectra of monomers EDOT and DTT show the absorption maxima peaks at 257 nm and 289 nm, respectively in the MeCN solution (Fig. S2†). To understand the relative consumption of monomers EDOT and DTT units in solutions used for electro-copolymerization, UV-visible absorption spectra of the solutions before and after electrochemical deposition of copolymers were recorded. All the proposed feed ratios of EDOT and DTT (2 : 1, 1 : 1 and 1 : 2) were used for the preparation of three different solutions in MeCN. Fig. 2 represents the absorption spectra of monomers solution with the different feed ratios of EDOT : DTT as 2 : 1, 1 : 1 and 1 : 2, respectively before and after the electro-copolymerization process. It was observed that the absorption spectra show pronounced differences by varying the feed ratios in the copolymerization mixtures. The monomers solution prepared with a 2 : 1 composition, having a higher concentration of EDOT shows a well-defined peak of EDOT and another peak for DTT, which indicates the occurrence of both EDOT and DTT units in the solution. While the solution having a 1 : 1 ratio of EDOT : DTT shows a less intense peak for EDOT, whereas, in a 1 : 2 ratio, the EDOT peak almost diminishes. Correspondingly, the characteristic peak of DTT gets enhanced as the ratio of DTT units increases in the solution. Moreover, the absorption spectra before and after the electro-copolymerization process reveal that the copolymers have maintained the composition of both monomer units as the feed ratio used for copolymerization. However, discrepancies have been found as the slight difference in the absorption spectra before and after the electro-copolymerization process. Further, it is worth noticing that the spectral change upon monomers incorporation does not indicate any chemical changes. Therefore, this shows that no chemical reaction takes place between EDOT and DTT and does not have any influence on the molecular structure during the electrochemical deposition of copolymer films.

### 3.3 FTIR spectra

**Fig. 3:**
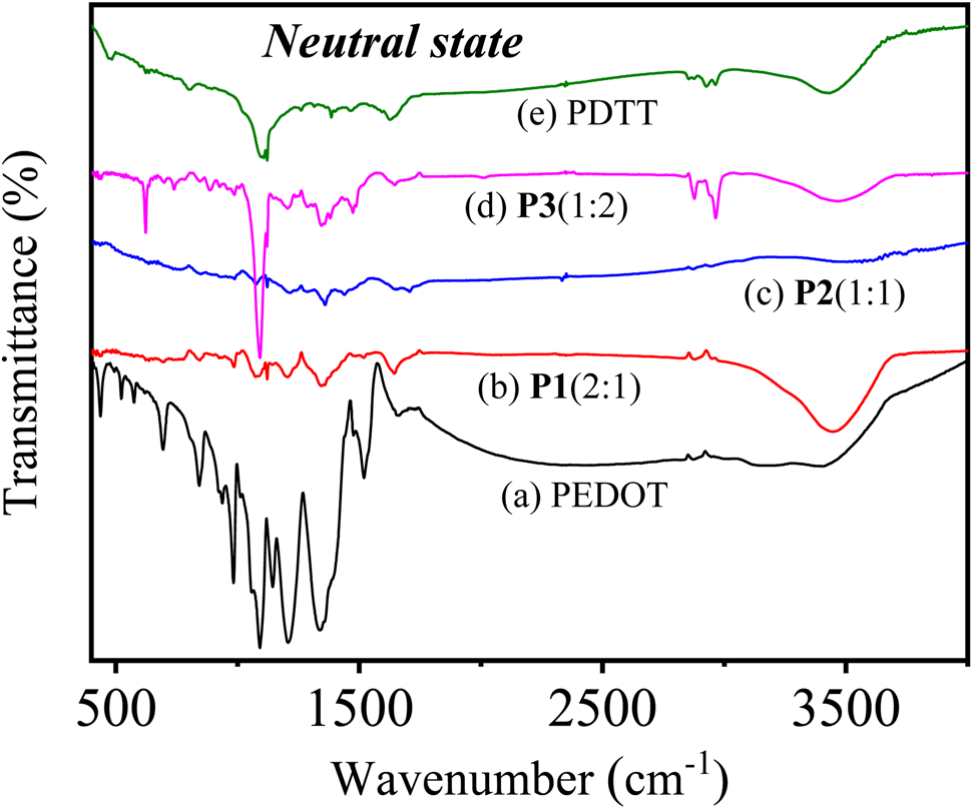
FTIR spectra of (a) PEDOT, (b) P1(2 : 1), (c) P2(1 : 1), (d) P3(1 : 2) and (e) PDTT in the neutral form.

To verify the presence of PEDOT as well as PDTT in the obtained copolymer films, FTIR spectroscopy were carried out. For FTIR investigation, copolymers P1(2 : 1), P2(1 : 1) and P3(1 : 2) were blended with anhydrous KBr and pressed into a pellet by hydraulic press for the measurement. For comparison purposes, FTIR spectra of their homopolymers PEDOT and PDTT were also recorded and is represented in Fig. S3(i).†Fig. 3 presents the FTIR spectra of PEDOT, P1(2 : 1), P2(1 : 1), P3(1 : 2) and PDTT in the neutral state. As seen from the spectrum of pure PEDOT in the neutral state [Fig. 3a], the bands at 1658, 1518, 1470 and 1338, 1210 cm^−1^ are attributed to the stretching vibration modes of C=C and C–C in the thiophene rings, respectively.^24,34,45^ While, the bands at 1144 and 1090 cm^−1^ originate from the stretching modes of C–O–C in the ethylenedioxy group^46,47^ and the bands at 982, 935, 842 and 695 cm^−1^ are assigned to the vibration from C–S bond in the thiophene ring of PEDOT.^48,49^ In the spectrum of pure PDTT [Fig. 3e], the vibration of C–S bond is reflected at 803 cm^−1^ and the bands at 1095 and 1126 cm^−1^ correspond to the C–S–C bond stretch. Whereas, the vibration of C=C and C–C are observed at ≈1380, 1465 and 1630 cm^−1^, respectively.

Compared with the corresponding homopolymers, the FTIR spectra of the neutral state of three copolymers P[EDOT-*co*-DTT] in Fig. 3(b–d) exhibits the above-mentioned characteristic bands of PEDOT and PDTT, suggesting the presence of both EDOT and DTT units in the formation of copolymers. Next, it is evident that the FTIR spectrum of P1(2 : 1) resembles more with pure PEDOT due to the higher ratio of EDOT units in the polymer matrix. As the feed ratio of EDOT is decreased in P2(1 : 1), the bands of PEDOT become less intense and the spectrum depicts broad bands with intermediate characteristics bands of both the homopolymers. On the other hand, in P3(1 : 2) a sharp peak appears at 1090 cm^−1^ which corresponds to C–S–C bond of PDTT, which indicates that the resulting copolymer film contains a higher ratio of DTT unit. These observations confirm that diverse feed ratios of monomers have resulted in the formation of three different copolymers, indicating the significant impact of feed ratios of monomers. Moreover, the resemblance of the obtained FTIR spectrum of all three copolymers with homopolymers indicates that copolymers have been successfully prepared *via* the electro-copolymerization method. Despite the difficulty of the detailed quantitative analysis due to the broadness and peak overlaps, the resulting FTIR absorption spectra clearly show that the peak intensity at correlating C–S stretch increases with the incorporation of more DTT units. Further, similar observations have been found in the oxidized state of the homopolymers and copolymers and are represented in Fig. S3(ii),† which confirms the corresponding polymers are copolymers, not the blend of PEDOT and PDTT.

### 3.4 Raman spectra

**Fig. 4:**
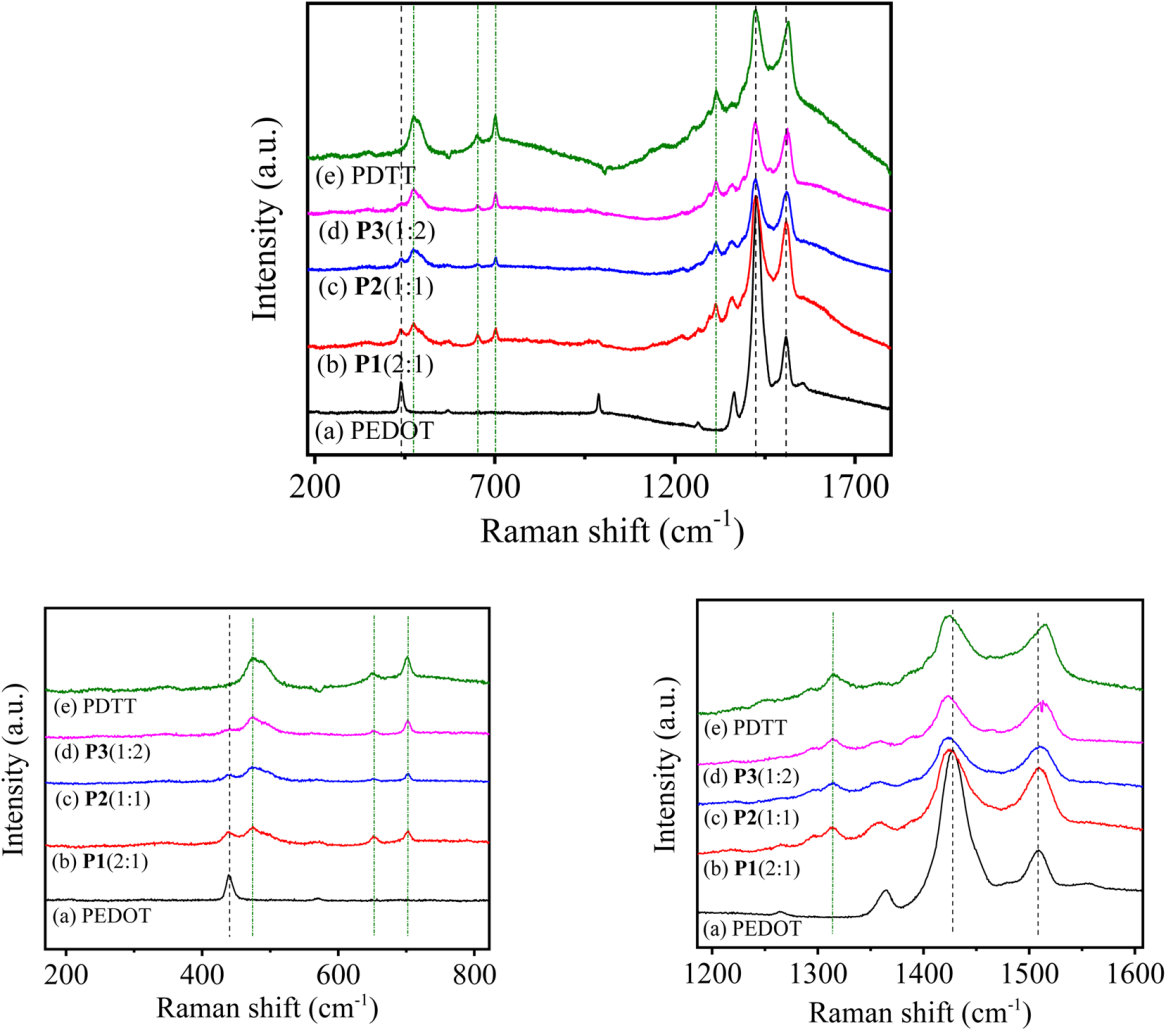
Raman spectra of (a) PEDOT, (b) P1(2 : 1), (c) P2(1 : 1), (d) P3(1 : 2) and (e) PDTT. Growth condition: polymers were electrodeposited potentiodynamically on ITO-coated glass substrate at 100 mV s^−1^ in 0.1 M TBAClO_4_/MeCN solution.

Raman spectroscopy was performed on electrodeposited copolymer films to further examine the composition and structure of P[EDOT-*co*-DTT] with different feed ratios of monomers EDOT and DTT units. Consequently, as-prepared thin films of different copolymers *i.e.*, P1(2 : 1), P2(1 : 1) and P3(1 : 2) on ITO-coated glass were directly subjected for the characterization. The spectral changes upon dissimilar monomers incorporation have been interpreted by correlating the band characteristics of the resultant copolymers with individual homopolymers (PEDOT and PDTT). Thus, the Raman spectra of homopolymers (PEDOT and PDTT) were also recorded under similar conditions. Fig. 4 represents the Raman spectra of PEDOT, P1(2 : 1), P2(1 : 1), P3(1 : 2) and PDTT, respectively from the wavenumber 180 to 1800 cm^−1^. The homopolymer PEDOT shows strong Raman bands near 438, 985, 1364, 1427 (very strong), 1508 cm^−1^ along with weak bands around 567, 1265 cm^−1^. While pure PDTT spectra present strong bands at 475, 1423, 1515 cm^−1^ along with medium bands around 650, 700, and 1315 cm^−1^. In the expanded spectra (1200–1600 cm^−1^, Fig. 4) of pure PEDOT, a strong peak at ∼1427 and 1507 cm^−1^ is the characteristics peak of symmetric and asymmetric C_α_ = C_β_ stretching, respectively with a shoulder peak at approximately 1363 cm^−1^, which corresponds to C_β_ = C_β_ stretching.^50,51^ Whereas, pure PDTT exhibits most prominent peaks at 1423 (symmetric C_α_ = C_β_ stretching), 1515 (asymmetric C_α_ = C_β_ stretching) and 1315 cm^−1^ (C_β_ = C_β_ stretching).

On comparing the different copolymers spectra with the homopolymers, copolymers P[EDOT-*co*-DTT] exhibit similar peak patterns of both the homopolymer PEDOT and PDTT, indicating the presence of both EDOT and DTT units in the resultant copolymers, formed with different feed ratios of monomers. It was observed that the spectra of the obtained copolymers show pronounced differences by varying the monomers compositions. For instance, in the P1(2 : 1) spectrum in Fig. 4b, all the vibrational modes are shifted to a lower frequency compared to homopolymer PEDOT, which is expected because of the incorporation of DTT units in the copolymer. The downshift of the C_α_ = C_β_ peak observed in copolymer films from 1426 [in P1(2 : 1)] to 1422 cm^−1^ [in P3(1 : 2)], indicates that the effective conjugation length has been increased, which is consistent with the increased number of DTT units in P3(1 : 2). The C_β_ = C_β_ peaks of copolymers, however, were shifted to ≈1358 cm^−1^ for P1(2 : 1) and ≈1357 cm^−1^ for P2(1 : 1) and P3(1 : 2). Further, no significant shifts were observed in expanded spectra of polymers ranging wavenumber from 180 to 820 cm^−1^. The vibrational assignment from the Raman spectra of P[EDOT-*co*-DTT] copolymers support the wavenumber assignment interpreted from the FTIR of the copolymers. Hence, from the FTIR and Raman spectra, we proposed that the P[EDOT-*co*-DTT] copolymer with three different compositions has been electrodeposited successfully on the ITO surface.

### 3.5 Elemental analysis

To quantitatively investigate the atomic compositions of the resultant copolymer films prepared from various feed ratios of EDOT and DTT, EDX analyses were performed. Both the neutral and oxidized forms of three different P[EDOT-*co*-DTT] films were examined by the EDX test. Table S2† depicts the field image and its corresponding EDX maps of three main peaks (C, O and S atoms) for P1(2 : 1), P2(1 : 1) and P3(1 : 2), respectively in neutral as well as in oxidized state. The summarized atomic composition derived from EDX data is represented in Table 1, suggesting the formation of different copolymers with various compositions of the monomers. On moving from the EDX spectrum of P1(2 : 1) to P3(1 : 2), a significant increase in sulphur peak with atomic composition increasing from 8.91% to 14.41% in neutral form and 11.21% to 13.64% in oxidized form was observed. Simultaneously, the decrease in oxygen peak (17.35% to 15.23% in neutral and 23.41% to 20.42% in oxidized state), confirms the incorporation of more DTT units in P3(1 : 2), as compared to P1(2 : 1). Whereas, from the EDX spectrum of P2(1 : 1), the intermediate outcomes were observed for sulphur and oxygen content, which is consistent with the feed ratio of 1 : 1 for EDOT and DTT. These observations show that copolymers P[EDOT-*co*-DTT] with different feed ratios of EDOT and DTT were successfully electrodeposited on the ITO-coated glass surface.

**Table 1:** Summarized atomic% composition of P[EDOT-*co*-DTT] copolymer films in the neutral and oxidized form prepared from different feed ratios of EDOT and DTT

P[EDOT-*co*-DTT]	Elements of P[EDOT-*co*-DTT]					
	In neutral form			In oxidized form		
	C%	O%	S%	C%	O%	S%
P1(2 : 1)	73.74	17.35	8.91	65.38	23.41	11.21
P2(1 : 1)	72.79	16.11	11.10	74.26	16.73	9.01
P3(1 : 2)	70.36	15.23	14.41	65.94	20.42	13.64

### 3.6 Redox properties of P[EDOT-*co*-DTT] copolymer films

**Fig. 5:**
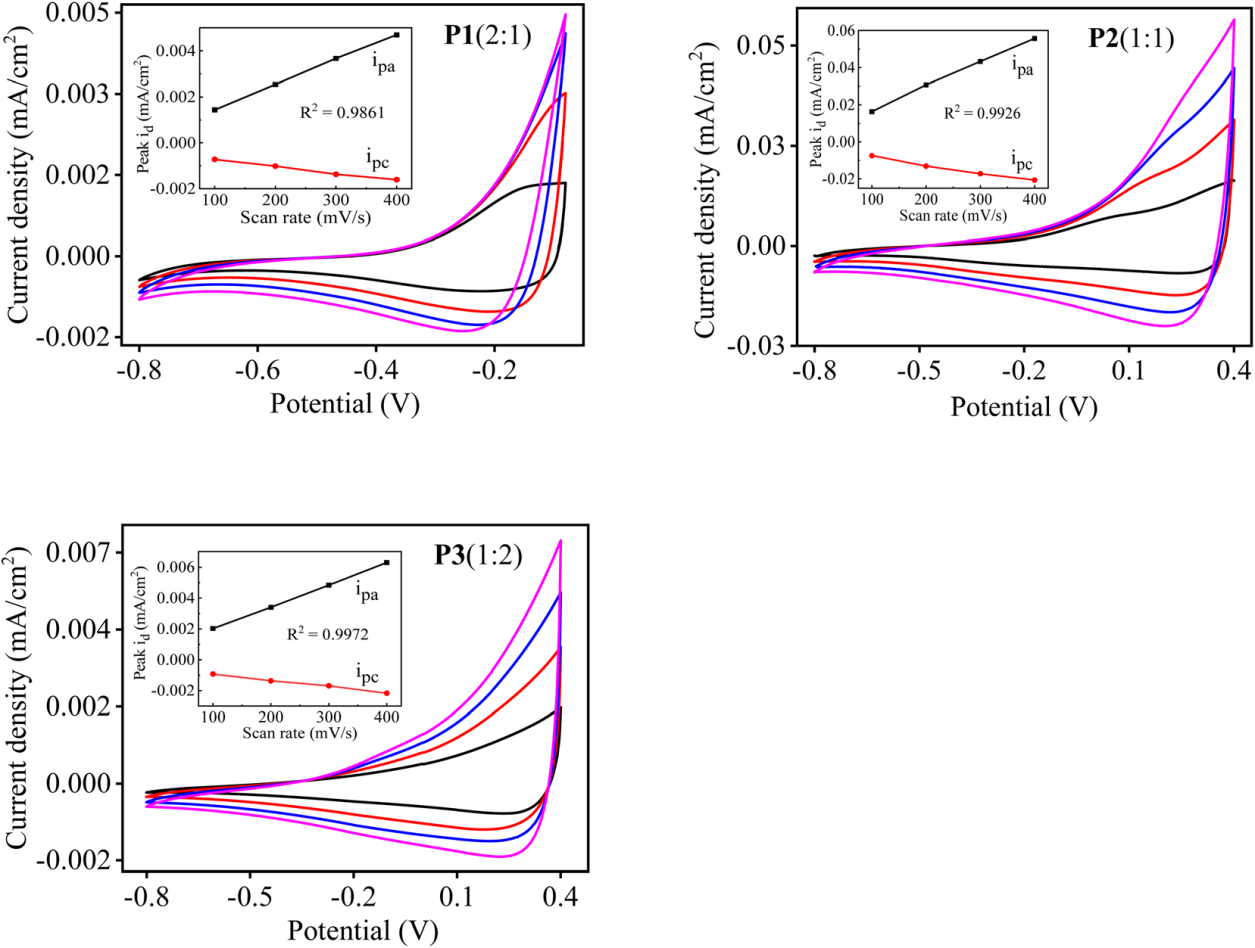
CV measurements of the electrodeposited copolymers films P1(2 : 1), P2(1 : 1) and P3(1 : 2) on Pt working electrode in a monomer-free electrolytic solution (0.1 M TBAClO_4_/MeCN) at the potential scan rate of 100, 200, 300 and 400 mV s^−1^ (*vs.* Ag/Ag^+^). Each inset shows the plot of peak current density as a function of the scan rate.

The electrochemical behaviour of the obtained copolymer films, P1(2 : 1), P2(1 : 1) and P3(1 : 2) were investigated by CV at the different scan rates of 100, 200, 300 and 400 mV s^−1^ in a single compartment three-electrode cell. P[EDOT-*co*-DTT] with various feed ratios of EDOT and DTT as 2 : 1, 1 : 1 and 1 : 2, respectively were electrodeposited on the Pt electrode by sweeping the potential from −0.8 to 1.5 V for four cycles. For CV measurements, the polymer-coated Pt was used as a working electrode, Ag wire as a reference and Au wire as a counter electrode in a monomer-free solution of MeCN containing 0.1 M TBAClO_4_. Fig. 5 shows the cyclic voltagramms of the different copolymers, *i.e.*, P1(2 : 1), P2(1 : 1) and P3(1 : 2), respectively. All three copolymer films show a reversible redox process between the anodic and cathodic peak potentials at different scan rates, suggesting a typical electrochemical behaviour of conjugated polymers. It was observed that the copolymers P[EDOT-*co*-DTT] prepared with different feed ratios show diverse electrical properties. For example, the oxidation onset potential (*E*^ox^_onset_) of the copolymers were found to be −0.41, −0.21 and −0.16 V corresponding to P1(2 : 1), P2(1 : 1) and P3(1 : 2), respectively. For comparison purposes, the CV of homopolymers were also recorded under similar conditions and is presented in the ESI Fig. S4.† It is evident that *E*^ox^_onset_ values of the copolymers are the intermediate values of the homopolymers, PEDOT (−0.62 V) and PDTT (0.21 V), which shows that the different copolymers of P[EDOT-*co*-DTT] have well-defined and intermediate redox properties. Further, the *E*^ox^_onset_ values were used to calculate the experimental HOMO energy level of the polymers and are summarized in Table 2. Compared to homopolymers, the HOMO energy level of the prepared copolymer films was found to be in between PEDOT and PDTT. The HOMO values of P[EDOT-*co*-DTT] were calculated to be −3.99, −4.19 and −4.24 eV for P1(2 : 1), P2(1 : 1) and P3(1 : 2), respectively. This interprets that different feed ratios of monomers have a significant effect on the HOMO level of the polymers and thus by varying the feed ratios, redox properties can be tuned for various applications.

**Table 2:** Redox properties of the copolymers P[EDOT-*co*-DTT] and homopolymers PEDOT and PDTT obtained from CV measurements

Polymers	*E* ^ox^_onset_ (V)	HOMO^a^ (eV)	*E*_pa_ (V)	*E*_pc_ (V)	*E* ^b^_1/2_ (V)
PEDOT	−0.77	−3.63	0.11	−0.61	−0.25
P1(2 : 1)	−0.41	−3.99	−0.13	−0.19	−0.16
P2(1 : 1)	−0.21	−4.19	0.27	0.22	0.24
P3(1 : 2)	−0.16	−4.24	0.37	0.26	0.32
PDTT	0.21	−4.61	0.96	0.82	0.89

a HOMO = −(4.8 − *E*_Fc/Fc+_ + *E*^ox^_onset_) eV, where *E*_Fc/Fc+_ is found to be 0.4 V *vs.* Ag/Ag^+^ and *E*^b^_1/2_ is the half-wave potential of the first oxidation and reduction process and is calculated as *E*_1/2_ = (*E*_pa_ + *E*_pc_)/2.

**Fig. 6:**
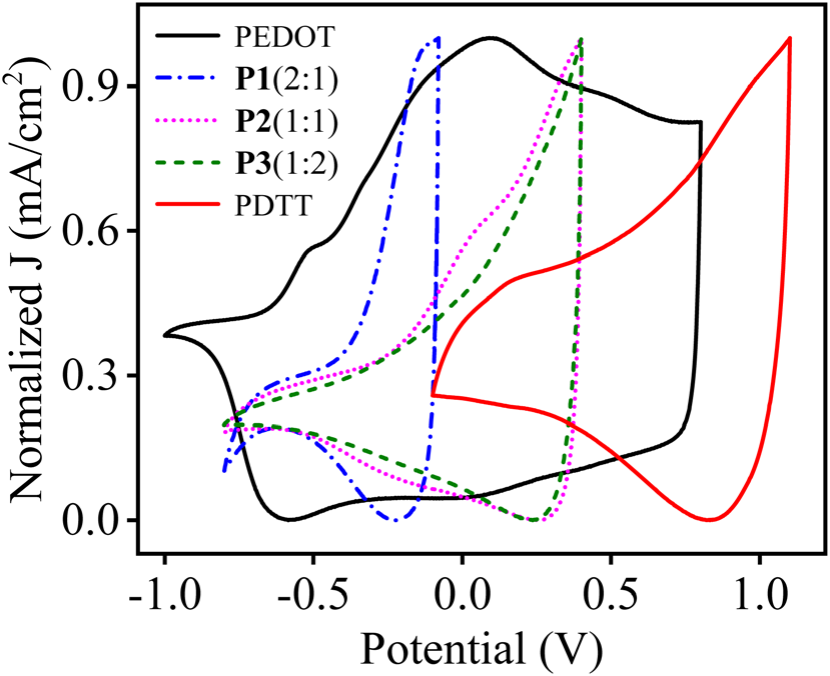
Normalised CV curves of PEDOT, P1(2 : 1), P2(1 : 1), P3(1 : 2) and PDTT, respectively at the scan rate of 100 mV s^−1^.

For a better understanding of the influence of monomers feed ratio on the electrical properties of prepared polymers, CV curves of homopolymers and copolymers were drawn together. Fig. 6 depicts the normalized CV curves of PEDOT, P1(2 : 1), P2(1 : 1), P3(1 : 2) and PDTT, respectively at the scan rate of 100 mV s^−1^. It is worth noticing that redox peaks of P1(2 : 1) which contains more EDOT units is nearer to PEDOT and as the concentration of EDOT decreases in P2(1 : 1) and even less in P3(1 : 2), a positive shift of peaks was observed. The positive shift of P3(1 : 2) is due to more DTT units incorporated into the copolymer chain, which correlates well with the elemental analysis results for P3(1 : 2).

Next, in all three copolymers, the peak current density response increases with an increase in the scan rate. The anodic peak potential (*E*_pa_) and cathodic peak potential (*E*_pc_) show a linear dependence as a function of scan rate, as depicted in the inset graph of Fig. 5. This also demonstrates that the electrochemical process of the copolymers is reversible and not diffusion-limited even at higher scan rates.^52^ Additionally, the *E*_pa_ and *E*_pc_ were used to determine the half-wave potential (*E*_1/2_) of the resultant copolymers. As can be seen in Fig. 5, when the feed ratio is 2 : 1, the copolymer P1(2 : 1) shows *E*_pa_ at 0.13 V and *E*_pc_ at 0.19 V at the scan rate of 100 mV s^−1^. As the feed ratio is changed to 1 : 1, the copolymer P2(1 : 1) shows *E*_pa_ and *E*_pc_ at 0.27 and 0.22 V, respectively. When the feed ratio becomes 1 : 2, the copolymer P3(1 : 2) presents *E*_pa_ at 0.37 V and *E*_pc_ at 0.26 V. Based on this, *E*_1/2_ values of each copolymer were calculated and is summarized in Table 2. Compared to homopolymers PEDOT (*E*_1/2_ ∼ 0.09 V) and PDTT (*E*_1/2_ ∼ 0.86 V), the copolymers P[EDOT-*co*-DTT] have in-between *E*_1/2_ values, which confirms the intermediate electrical characteristics of the resultant copolymers. This further depicts the importance of the feed ratio of monomers during the electropolymerisation process.

### 3.7 Absorption and spectroelectrochemical properties

**Fig. 7:**
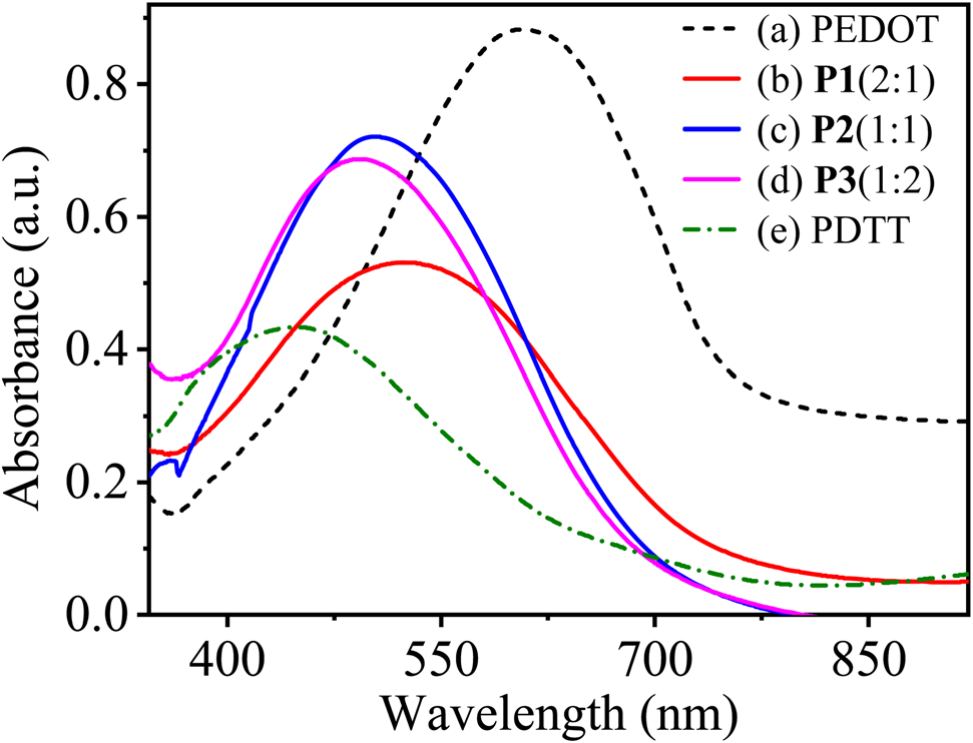
UV-visible absorption spectra of (a) PEDOT, (b) P1(2 : 1), (c) P2(1 : 1), (d) P3(1 : 2) and (e) PDTT electrodeposited on ITO-coated glass in the neutral state.

UV-visible absorption spectra of homopolymers (PEDOT and PDTT) and their copolymers P[EDOT-*co*-DTT] prepared with different feed ratios of EDOT and DTT (2 : 1, 1 : 1 and 1 : 2) on ITO-coated glass slides were recorded over the wavelength range from 300 to 900 nm. Fig. 7 depicts UV-visible spectra of homopolymers and the copolymers P1(2 : 1), P2(1 : 1) and P3(1 : 2) in the neutral state. The obtained copolymers show one strong peak with maximum absorbance wavelength (*λ*_max_) at 520, 505 and 490 nm corresponding to P1(2 : 1), P2(1 : 1) and P3(1 : 2), respectively and are attributed to the π–π* electronic transition from the valence band to the conduction band in conjugated polymers. In contrast, the homopolymers PEDOT and PDTT show characteristic absorption peaks at about 610 and 440 nm, respectively. The diverse and in-between *λ*_max_ values of P[EDOT-*co*-DTT] films show the significance of the feed ratio of monomers during the electropolymerisation process. It is worth noticing that compared to pure PEDOT, all the copolymers exhibit a continuous blue shift of the absorption peak as the feed ratio of EDOT : DTT decreases, implying the increase of DTT units in the polymer chain, and further confirming the occurrence of copolymerization and as well by the results of elemental analysis.

**Fig. 8:**
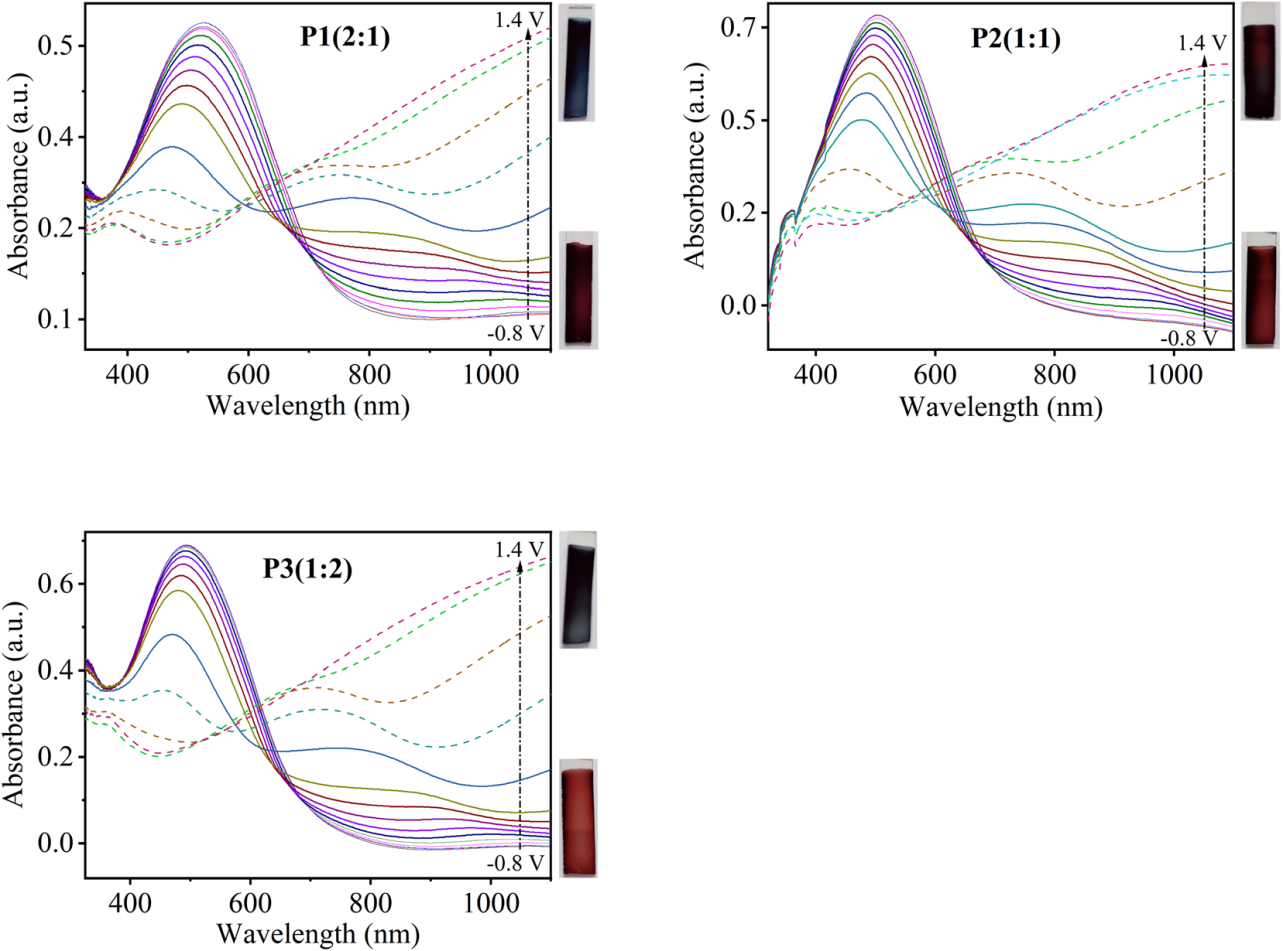
Spectroelectrochemical spectra of the copolymers P1(2 : 1), P2(1 : 1) and P3(1 : 2), prepared on ITO-coated glass as a function of applied potential between −0.8 to 1.4 V in 0.1 M TBAClO_4_/MeCN; reference electrode: Ag wire (Ag/Ag^+^); counter electrode: Au wire.

To further examine the effect of monomers feed ratio on the optoelectronic properties of the prepared copolymer films, *in situ* spectroelectrochemical investigations were performed. *In situ* UV-visible absorption spectra of copolymer films deposited on ITO-coated glass slides were recorded in the potential range from −0.8 to 1.4 V in a monomer-free solution of 0.1 M TBAClO_4_ in MeCN. The change in absorbance spectra of P1(2 : 1), P2(1 : 1) and P3(1 : 2) as a function of different applied potentials is shown in Fig. 8. In neutral state (undoped form, −0.8 V), P[EDOT-*co*-DTT] films exhibit a broad peak at their respective *λ*_max_ (520, 505 and 490 nm for P1(2 : 1), P2(1 : 1) and P3(1 : 2), respectively) which relates to π–π* electronic transition. As the potential is increased, the films get oxidized and the intensity of π–π* transition decreases and a new absorption peak appears around 700 nm and 1050 nm which is due to the formation of polaron and bipolarons, respectively. P[EDOT-*co*-DTT] formed with a different feed ratio of EDOT : DTT show significantly different isosbestic points when the potential was applied up to 0.8 V as illustrated in Fig. 8.

It is well-known that PEDOT shows blue and transmissive grey colour in neutral and oxidized states, respectively. While PDTT shows red-orange in undoped form and blue-grey colour in doped form. Interestingly, it was found that the resultant copolymers show different colour in neutral as well as in oxidized state, compared to homopolymers, PEDOT and PDTT. Table S3† illustrates the photographs of copolymers P[EDOT-*co*-DTT] electrodeposited onto ITO-coated glass from various feed ratios of EDOT and DTT as 2 : 1, 1 : 1 and 1 : 2 in both neutral and oxidized states. P1(2 : 1) shows wine colour at −0.8 V and blue colour at 1.4 V while, P2(1 : 1) displays brick-red and brown colour at neutral and oxidized states, respectively. For P3(1 : 2), the colours are red-orange to blue-grey at the neutral and oxidized state. This interprets that different feed ratios of EDOT and DTT enrich the colour range of P[EDOT-*co*-DTT] copolymers, which could have significant applications in optoelectronic devices. The optical band-gap energy (*E*_g_) of the different copolymer films, P1(2 : 1), P2(1 : 1) and P3(1 : 2) were calculated to be 1.67 eV (*λ*_onset_ ∼ 740 nm), 1.71 eV (*λ*_onset_ ∼ 725 nm) and 1.75 eV (*λ*_onset_ ∼ 705 nm), respectively, which are higher than that of PEDOT (1.6 eV) and lower than PDTT (1.94 eV). This infers that electro-copolymerization with different feed ratios of EDOT and DTT has positively resulted in the tuning of bandgap energy of the resultant copolymers, which is suitable for applications like organic light light-emitting diodes and sensors. Table 3 summarizes the maximum absorption wavelength (*λ*_max_), low-energy absorption edge wavelength (*λ*_onset_), optical band-gap energy (*E*_g_), full width at half maxima (FWHM) of PEDOT, copolymers P1(2 : 1), P2(1 : 1), P3(1 : 2) and PDTT.

**Table 3:** The spectroelectrochemical data of PEDOT, P1(2 : 1), P2(1 : 1), P3(1 : 2) and PDTT

Polymers	*λ*_max_ a (nm)	*λ*_onset_ b (nm)	*E*_g_ c (eV)	FWHM^d^ (nm) [eV]
PEDOT	610	775	1.60	228 [0.81]
P1(2 : 1)	520	740	1.67	198 [0.92]
P2(1 : 1)	505	725	1.71	185 [0.91]
P3(1 : 2)	490	688	1.80	155 [0.79]
PDTT	440	640	1.94	111 [0.61]

a Wavelength at the maximum absorbance.

b Wavelength at the onset of π–π* electronic transition.

c Optical band gap calculated from the equation: *E*_g_ = 1240/*λ*_onset_.

d FWHM values of absorption peak in nm and values in bracket are in eV.

### 3.8 Morphological properties

**Fig. 9:**
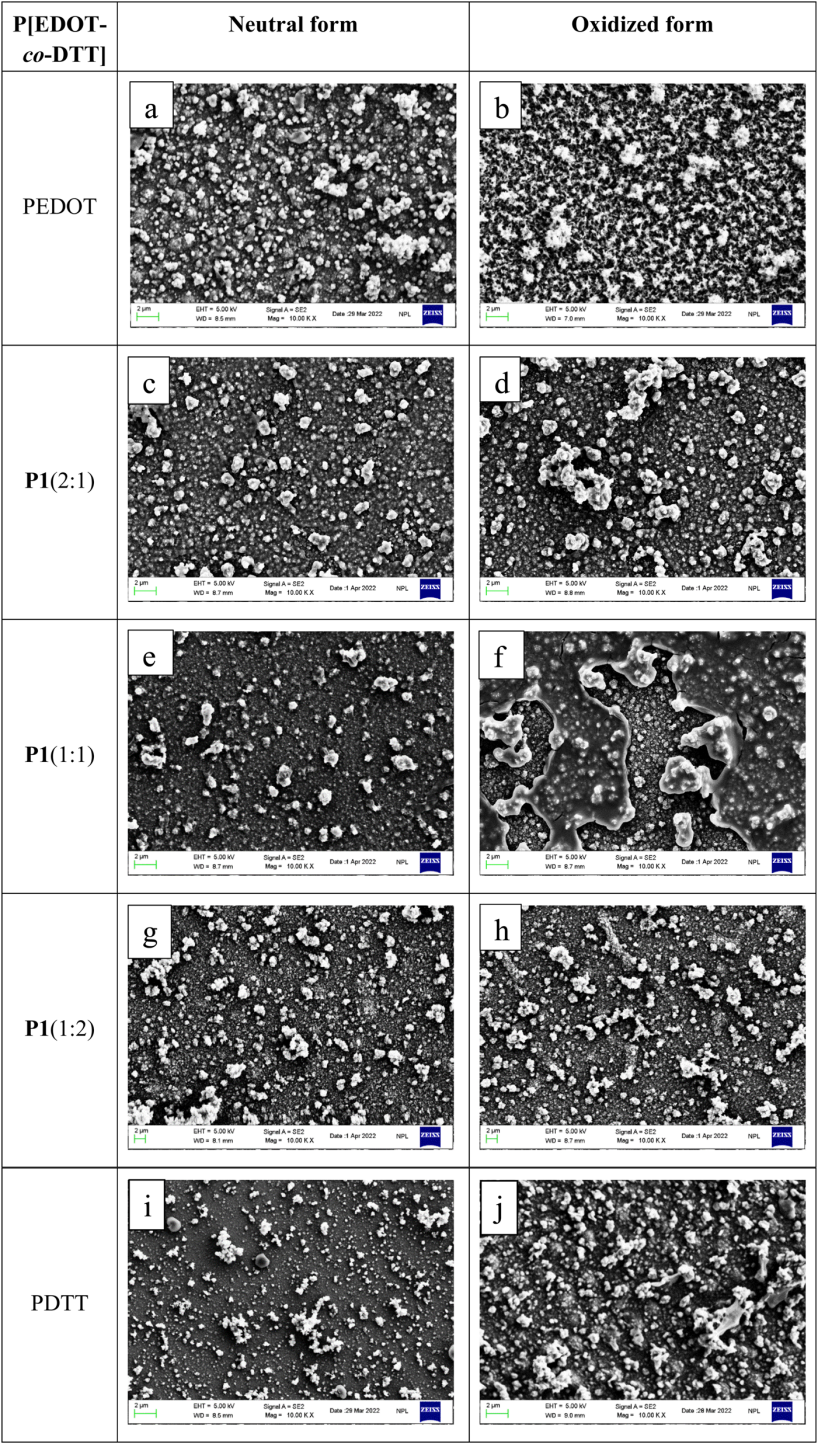
The FESEM micrographs of PEDOT (a, b), copolymer films P[EDOT-*co*-DTT] electrodeposited from different feed ratios of EDOT : DTT as 2 : 1 (c, d), 1 : 1 (e, f) and 1 : 2 (g, h) and PDTT (i, j) on the ITO surface. Images on the left-hand side (a, c, e, g and i) and right-hand side (b, d, f, h and j) are the neutral and oxidized form of the films, respectively.

To examine the surface morphology of the resultant polymers, FESEM analysis was performed in the neutral as well as in the oxidized state of the copolymer films. P[EDOT-*co*-DTT] copolymer films with various feed ratios of monomers (EDOT : DTT as 2 : 1, 1 : 1 and 1 : 2, respectively) were electrodeposited on ITO substrate by potentiodynamic method in 0.1 M TBAClO_4_/MeCN in the potential range −0.8 to 1.5 V. All the films were rinsed in acetonitrile to remove residual electrolytes, monomers and dried under nitrogen before analysis. For comparison purposes, FESEM analysis of homopolymers PEDOT and PDTT was also carried out. Fig. 9 represents the FESEM micrographs of PEDOT, P1(2 : 1), P2(1 : 1), P3(1 : 2) and PDTT. The images on the left side (a, c, e, g and i) show the neutral form, whereas, right-side (b, d, f, h and j) shows the oxidized form of the respective conjugated polymers. We found that PEDOT exhibits an accumulation of clusters of globules in a neutral state (Fig. 9a) and loose spongy network structure in oxidized form (Fig. 9b), which correlates well with the earlier reported results in organic solution.^53^ While neutral PDTT (Fig. 9i) shows a smooth surface with small-sized particles all over the surface and oxidized PDTT (Fig. 9h) depicts higher surface coverage with an ordered arrangement. It is worth noticing that the micrograph of copolymers reveals significantly different surface morphology from the two corresponding homopolymers. We have found that depending upon the monomers' feed ratios, the surface morphologies of copolymer films varied meaningfully both in neutral as well as in oxidized state. The copolymer P1(2 : 1) in the neutral form shows globules dispersed all over the surface, while the oxidized form shows smaller granules, which is very different from PEDOT. In contrast, the surface of P2(1 : 1) film displays non-uniform-sized particles distributed all over the surface both in neutral and oxidized states. P3(1 : 2) displays a homogeneous distribution as the neutral form reveals a porous and rough morphology, while the oxidized form, films revealed a more compact and rougher surface. These variations in the surface morphology of the different copolymers coincide well with the above-mentioned outcomes from CV (good redox activity) and spectroelectrochemistry.

### 3.9 Computational studies

**Fig. 10:**
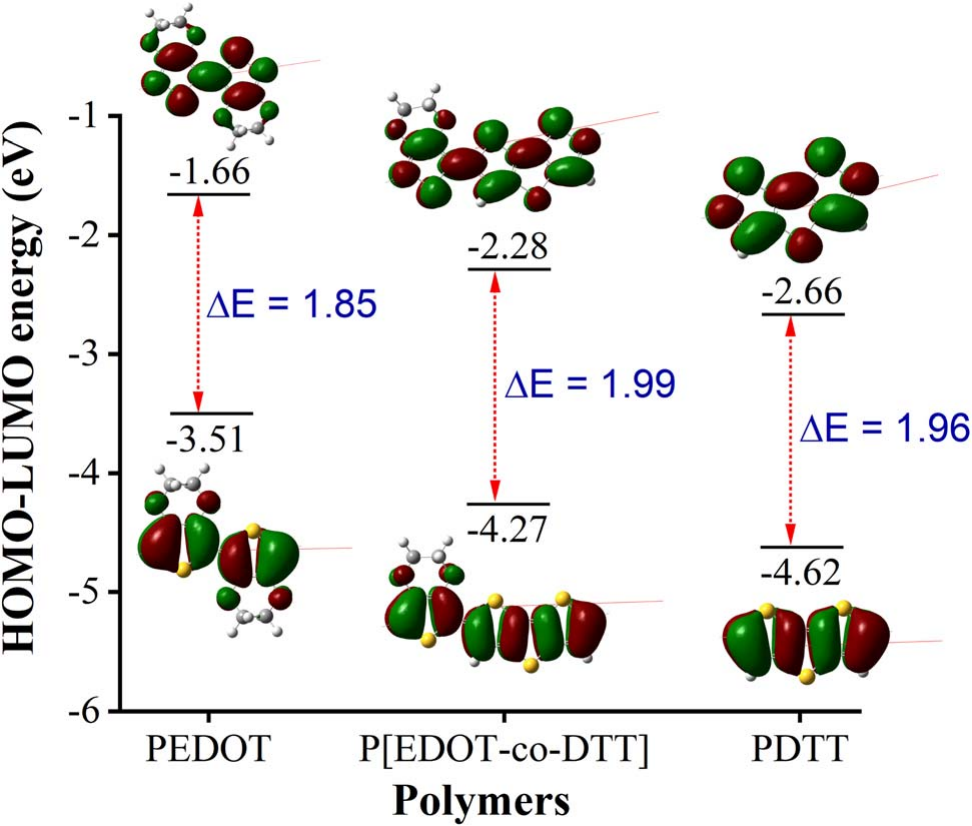
Optimized ground state frontier molecular HOMO and LUMO orbitals of PEDOT, P[EDOT-*co*-DTT] and PDTT.

Theoretically, the optical and electronic properties of the molecules were studied through density functional theory (DFT) with the help of electronic distribution patterns of Frontier Molecular Orbitals (FMOs). For DFT calculations, basis set 6-31G(d) was used with the B3LYP level of theory to analyse the effect of the basis set on optoelectronic properties which were further compared with our experimental results. The calculated value of HOMO, LUMO and band gap for the homopolymers and copolymer is illustrated in Table 4. The HOMO levels of homopolymers are at −3.51 eV for PEDOT and −4.62 eV for PDTT, whereas LUMO levels are at −1.66 and −2.66 eV for PEDOT and PDTT, respectively. It was found that the HOMO energy of P[EDOT-*co*-DTT] in a 1 : 1 ratio of EDOT and DTT is −4.27 eV, while LUMO is at −2.28 eV, which is in-between values of their corresponding homopolymers. Experimental HOMO energy values obtained from the onset of oxidation peaks in CV curves correlate well with the theoretical data for polymers, as illustrated in Table 4. If a correction value (Δ*E*) 0.02 eV is applied to correlate the experimental and theoretical values. The DFT optimized structure and electronic distribution patterns of the homopolymers, and copolymer P[EDOT-*co*-DTT] are shown in Fig. 10.

**Table 4:** Using PBC/B3LYP/6-31G(d) calculated basis set HOMO, LUMO and HOMO–LUMO gap, along with the experimental band gap extracted from the absorption spectra

Polymers	HOMO (eV)	LUMO (eV)	*E*_g(calc.)_ a (eV)	*E*_g(exp.)_ b (eV)	Δ*E*^c^ (eV)
PEDOT	−3.51	−1.66	1.85	1.60	0.25
P[EDOT-*co*-DTT]	−4.27	−2.28	1.99	1.71	0.28
PDTT	−4.62	−2.66	1.96	1.94	0.02

a Band gap calculated from *E*_g(calc)_ = (LUMO – HOMO) eV.

b Experimental band gaps from the onset of the absorption spectra in the neutral state.

c Difference in energy is calculated by Δ*E* = *E*_g(calc.)_ − *E*_g(exp)_.

### 3.10 Electrochromic properties of P[EDOT-*co*-DTT] films

**Fig. 11:**
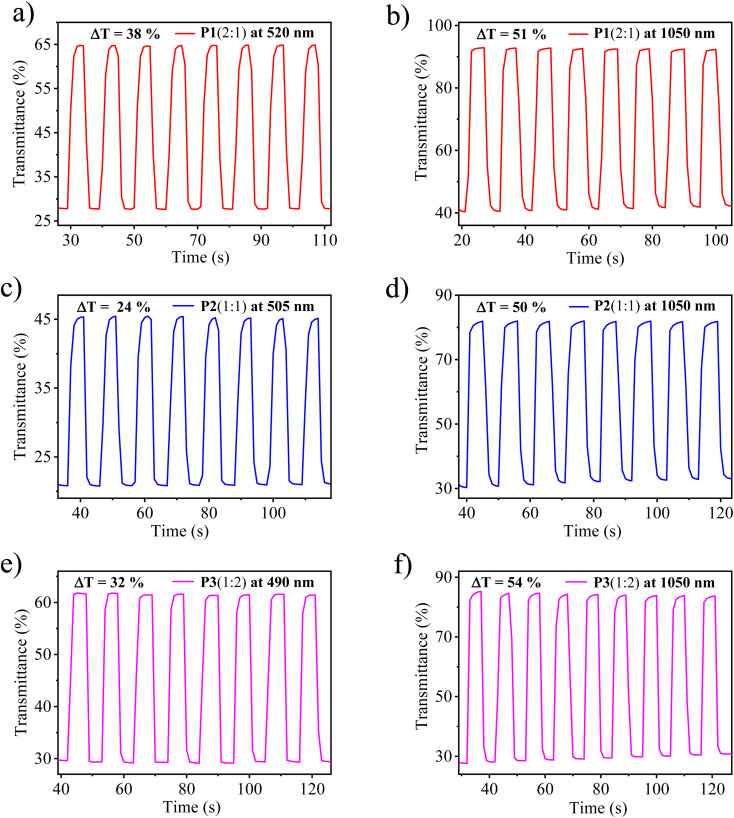
Transmittance spectra of copolymers (a and b) P1(2 : 1), (c and d) P2(1 : 1) and (e and f) P3(1 : 2) films at *λ*_max_ and 1050 nm by switching between −1.0 to 1.0 V (*vs.* Ag/Ag^+^) with the residence time of 5 s.

To investigate the switching property and ability to exhibit striking colour change between its neutral and oxidized state, electrochromic switching studies were carried out. Accordingly, we have considered the three different copolymers P1(2 : 1), P2(1 : 1) and P3(1 : 2) to determine the electrochromic characteristics such as optical contrast (Δ*T*%), response time and coloration efficiency (CE) for the different copolymer films. The copolymer films were electrodeposited on ITO and the double potential step chronoamperometry technique was used to test the switching ability between its neutral and oxidized state with a change in transmittance at a fixed wavelength. All the copolymer films were switched between −1.0 and 1.0 V at a regular interval of 5 s, and the percentage transmittance (% *T*) of the copolymers was at *λ*_max_ and 1050 nm. Considering the impact of feed ratios of monomers, the Δ*T*% between the redox states of the copolymers at their respective *λ*_max_ was calculated to be 38% (520 nm), 24% (505 nm) and 32% (490 nm) for P1(2 : 1), P2(1 : 1) and P3(1 : 2) respectively (Fig. 11a, c and e). The corresponding chronoamperograms showing current consumption during the electrochromic switching of 0.1 M MeCN/TBAClO_4_ electrolyte solution are shown in Fig. S5.† At the higher wavelength of 1050 nm, the Δ*T*% was found to be 51%, 50% and 54% for the corresponding copolymers P1(2 : 1), P2(1 : 1) and P3(1 : 2) and is shown in Fig. 11b, d and f, respectively. Conversely, the lower Δ*T*% were recorded for the homopolymers PEDOT as well as PDTT under similar conditions and is illustrated in the Fig. S6.† Next, the optical response time of P1(2 : 1) was found to be 1.8 s from reduced to oxidized state and 1.4 s from oxidized to reduced state at 520 nm. We have observed that the copolymer P3(1 : 2) has a fast response when compared with the P1(2 : 1) and P2(1 : 1), as represented in Table 5. The faster-switching response of P3(1 : 2) film may be attributed due to the faster dopant ion diffusion during the redox process, which is due to the introduction of more DTT units into the polymer backbone. Then, the coloration efficiency (CE) at the higher wavelength of 1050 nm was calculated to be 78.0, 105.1 and 122.8 cm^2^ C^−1^ for P1(2 : 1), P2(1 : 1) and P3(1 : 2), respectively. Consequently, P3(1 : 2) shows comparatively high CE values in comparison to P1(2 : 1) and P2 (1 : 1) for all the respective wavelengths, making them suitable for electrochromic devices such as smart windows, mirrors.

**Table 5:** Electrochromic performance of the various P[EDOT-*co*-DTT] films deposited on ITO-coated glass at *λ*_max_ and 1050 nm in 0.1 M TBAClO_4_/MeCN solution

Copolymers	Wavelength (nm)	Optical contrast Δ*T* (%)	Response time^a^ (s)		Injected charge density (μC cm^−2^)	CE^b^ (cm^2^ C^−1^)
			*τ*_oxi_	*τ*_red_		
PEDOT	610	28	2.4	2.1	3.85	63.9
	1050	39	2.2	2.2	3.15	65.8
P1(2 : 1)	520	38	1.8	1.4	5.28	72.4
	1050	51	2.1	2.1	4.50	78.0
P2(1 : 1)	505	24	1.6	1.4	4.68	70.7
	1050	50	1.9	1.6	3.89	105.1
P3(1 : 2)	490	32	1.6	1.2	4.07	80.7
	1050	54	1.5	1.1	3.64	122.8
PDTT	475	27	1.1	1.0	2.62	74.7
	1050	40	1.2	1.1	2.46	112.7

a Response time calculated at 90% of a full switch from neutral to oxidized state (*τ*_oxi_)and *vice versa* for *τ*_red_.

b Coloration efficiency (CE) is calculated by CE = log(*T*_oxi_/*T*_red_)/injected charge density, where *T*_oxi_ and *T*_red_ refer to transmittance in the oxidized and neutral state, respectively.

## 4. Conclusions

In summary, a straightforward methodology for the synthesis of complex conjugated materials from low-cost monomer units with controlled stoichiometric ratios has been developed. Thus, three different copolymers P[EDOT-*co*-DTT] films were deposited on ITO-coated glass using three different feed ratios of monomers *via* an electro-copolymerization method in 0.1 M TBAClO_4_/MeCN solution. The synthesized copolymers were well characterized by FTIR, Raman spectroscopy and UV-visible spectroscopy and confirmed that the obtained copolymers contain both EDOT and DTT units. EDX analysis shows that the ratio of EDOT and DTT units in copolymers changes as the feed ratio of EDOT and DTT varies during polymerization. We have observed that feed ratios of monomers significantly affect the electrical, optical and morphological properties of the copolymer films. The electrochemical and spectroelectrochemical properties of all three copolymer films were in between their parent homopolymers. Fine-tuning in the band gap with HOMO and LUMO energy levels was achieved by tailoring the feed ratios of monomers in the copolymerization mixture. The obtained copolymer films exhibit smooth morphology and tunable electrochromism, which can be extended for the fabrication of electrochromic devices. In addition, the DFT calculations supported our experimental findings. Thus, copolymers P[EDOT-*co*-DTT] can be considered as a good candidate for organic electronic applications such as organic photovoltaics, organic light-emitting diodes, *etc.* The copolymers display the property of tunable electrochromism with improved transmittance and redox color change between the neutral and oxidized states, hence finding the applications for electrochromic devices such as smart windows, mirrors, and displays. Further additional monomer units in a complex conjugated system for the tailoring of the next-generation optoelectronic materials are under process in our laboratory.

## Conflicts of interest

There are no conflicts to declare.

## Supplementary Material

RA-014-D3RA08729H-s001
