# Supplementary material for: Electrochemical copolymerization of 3,4-ethylenedioxythiophene and dithienothiophene: influence of feed ratio on electrical, optical and electrochromic properties
Source: RSC Adv. 2024 Apr 8;14(16):11334–49. doi: 10.1039/d3ra08729h (PMC11000263; doi:10.1039/d3ra08729h)
Supplement: RA-014-D3RA08729H-s001 [file RA-014-D3RA08729H-s001.pdf]

Electronic Supplementary Material (ESI) for RSC Advances.
This journal is © The Royal Society of Chemistry 2024

Supporting Information

Electrochemical copolymerization of 3,4-ethylenedioxythiophene and dithienothiophene: influence of feed ratio on electrical, optical and electrochromic properties

Rashi Kedia,[ab] Manisha Khatak,[ab] Manisha Balkhandia[ab] and Asit Patra*[ab]

[a]Photovoltaic Metrology Section, Advanced Materials & Device Metrology Division, CSIR-National Physical Laboratory, Dr K. S. Krishnan Marg, New Delhi-110012, India

[b]Academy of Scientific and Innovative Research (AcSIR), Ghaziabad-201002, India

*Corresponding Author: apatra@nplindia.org

Table S1. Molar feed ratio of EDOT and DTT used to prepare different copolymers.

**Copolymers P[EDOT-*co*-DTT]**	**Monomers feed ratio in the copolymerization mixture**	
	**EDOT**	**DTT**
**P1**(2:1)	2	1
**P2**(1:1)	1	1
**P3**(1:2)	1	2

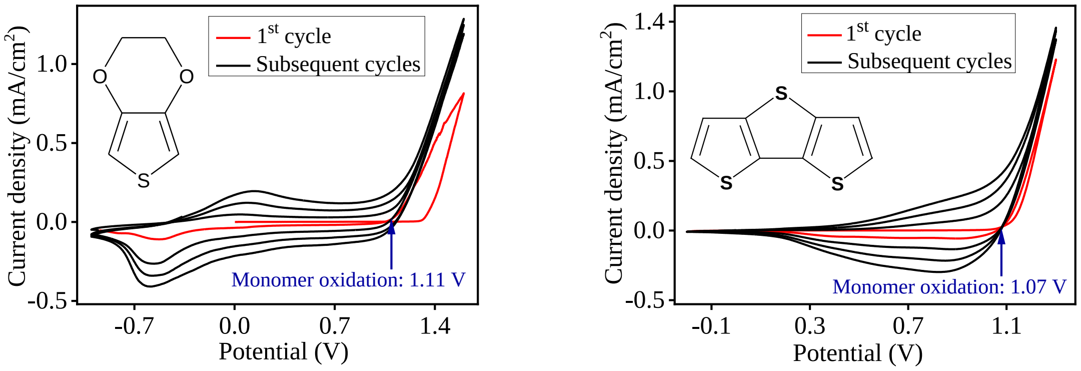

Figure S1. Electrocopolymerization curves of EDOT and DTT in 0.1 M TBAClO$_4$/MeCN solution to electrodeposit homopolymers PEDOT and PDTT at scan rate of 100 mV/s (*vs* Ag/Ag$^+$ wire, Fc/Fc$^+$ = 0.40 V).

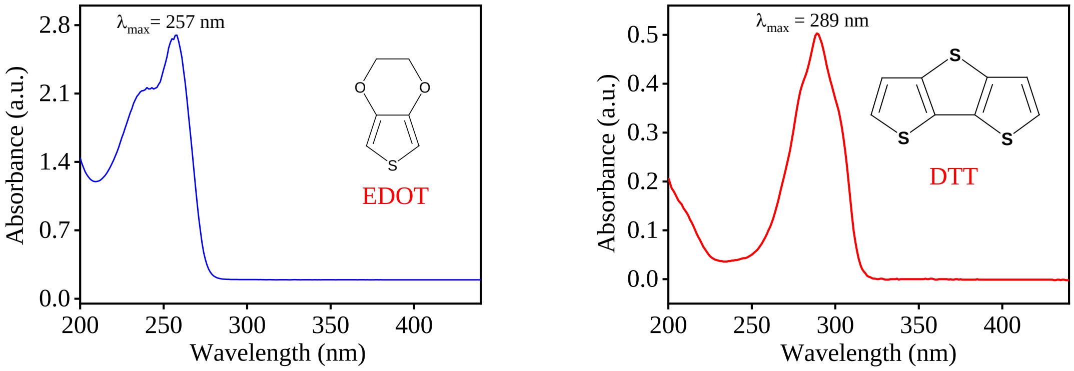

Figure S2. UV-visible absorption spectra of EDOT and DTT in acetonitrile solution.

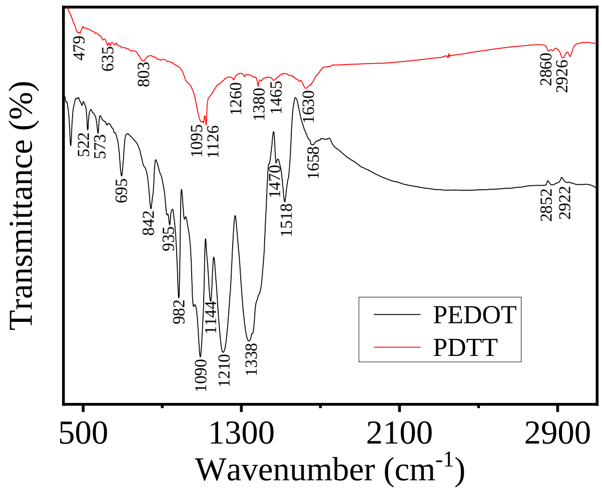

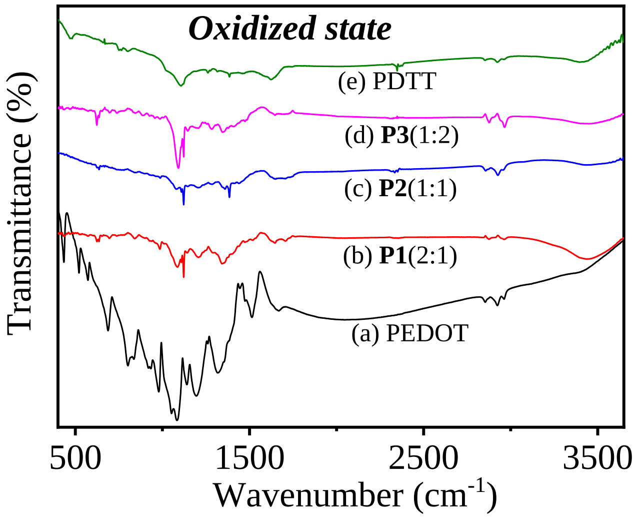

Figure S3. (i) FTIR spectra of PEDOT and PDTT. (ii) FTIR spectra of (a) PEDOT, (b) **P1**(2:1), (c) **P2**(1:1), (d) **P3**(1:2) and (e) PDTT in the oxidized form.

Table S2. Elemental analysis results: EDX spectrum and quantitative analysis of elemental composition of P[EDOT-*co*-DTT] copolymers obtained from various feed ratios of EDOT and DTT.

Copolymer	Neutral state	Oxidized state
**P1**(2:1)	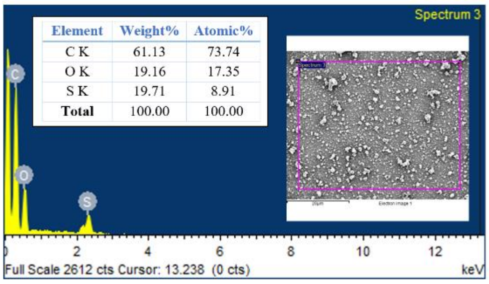	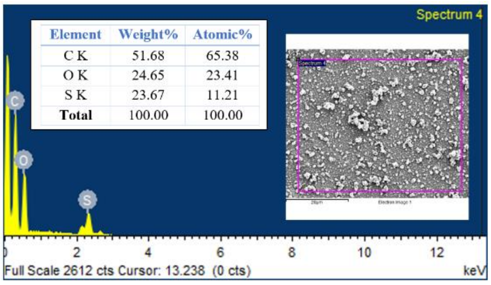
**P2**(1:1)	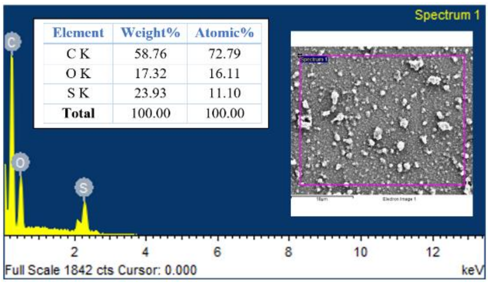	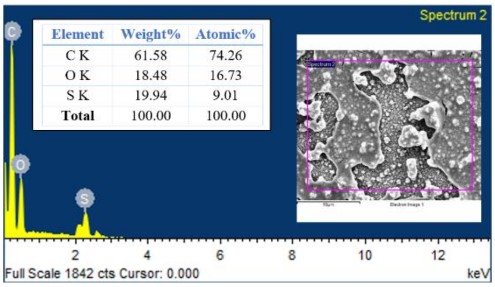

**P3**(1:2)	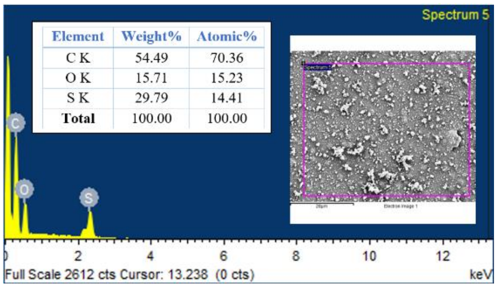	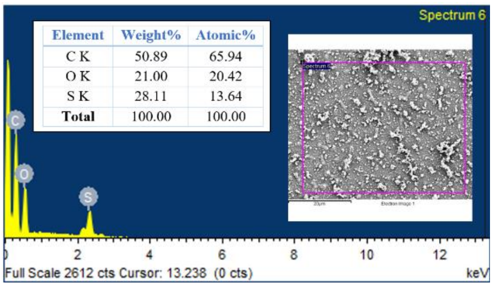

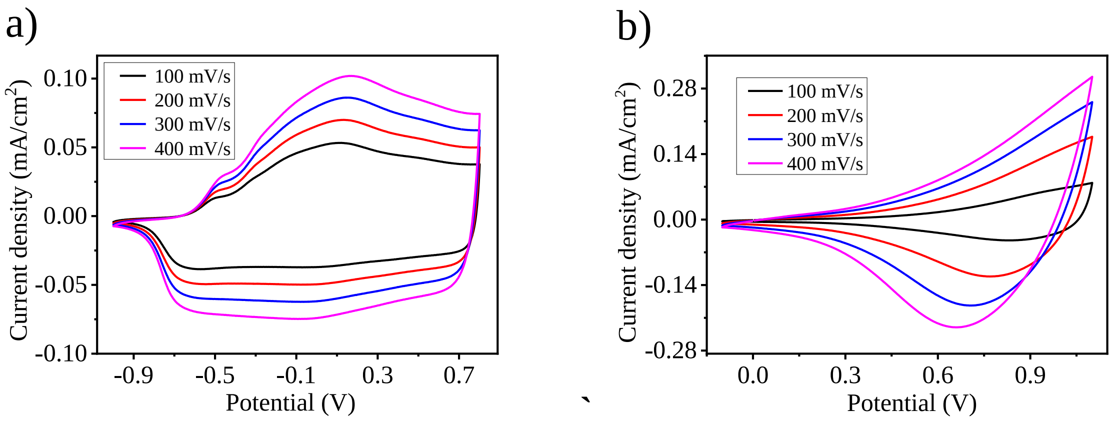

Figure S4. CV curves of PEDOT and PDTT on Pt working electrode in monomer-free electrolytic solution (0.1 M $TBAClO_4$/MeCN) at potential scan rate of 100, 200, 300 and 400 mV/s (*vs* Ag/Ag^+).

Table S3. Photographs of copolymers electrodeposited onto ITO electrode in various feed ratio of EDOT and DTT as 2:1, 1:1 and 1:2. Neutral form denotes the undoped form of the copolymers while oxidized form shows the doped condition.

**Copolymers**	**Colour of copolymers**	
	**Neutral**	**Oxidized**
**P1**(2:1)	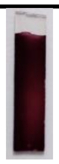	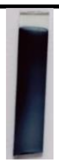

**P2**(1:1)	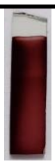	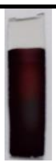
**P3**(1:2)	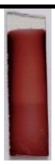	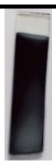

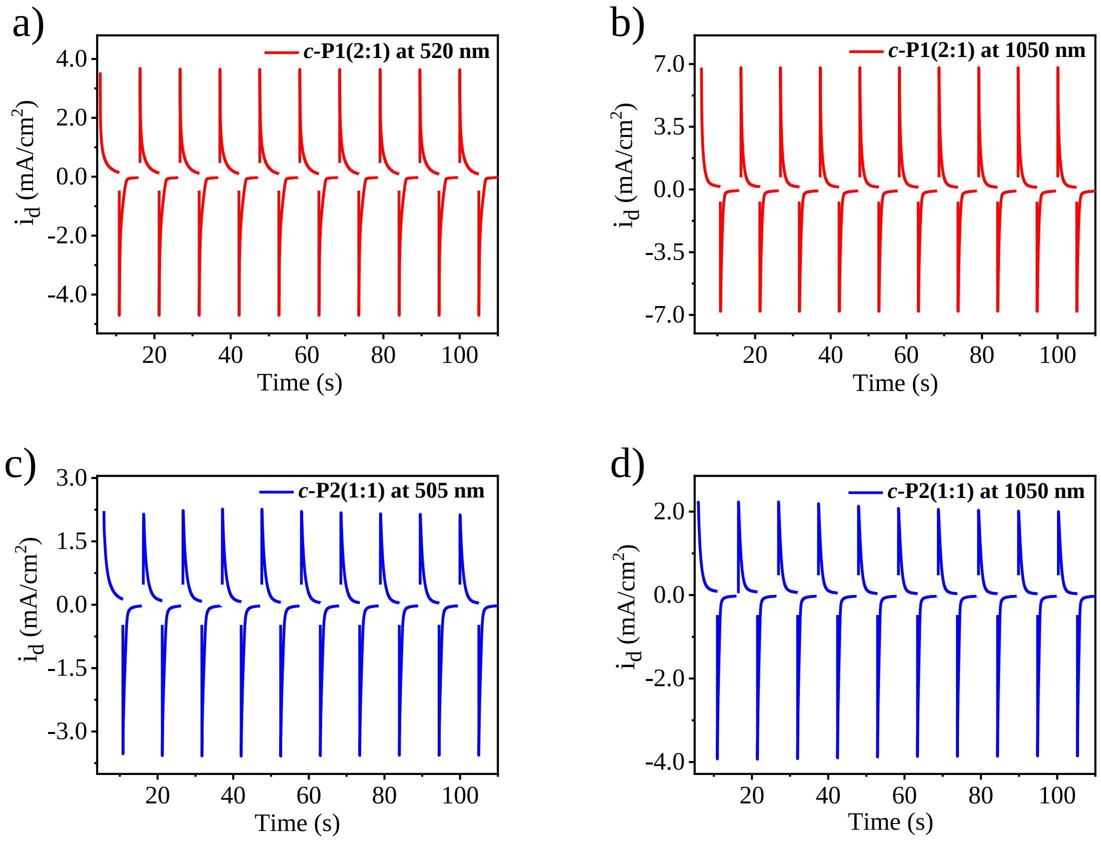
a)
c-P1(2:1) at 520 nm
i_d (mA/cm²)
Time (s)
b)
c-P1(2:1) at 1050 nm
i_d (mA/cm²)
Time (s)
c)
c-P2(1:1) at 505 nm
i_d (mA/cm²)
Time (s)
d)
c-P2(1:1) at 1050 nm
i_d (mA/cm²)
Time (s)

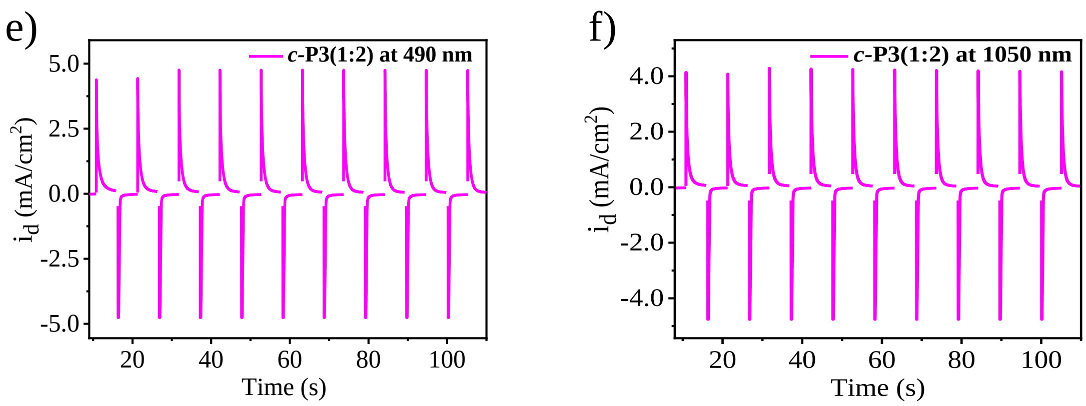

Figure S5. Chronoamperograms showing current consumption during the electrochromic switching of P[EDOT-*co*-DTT] in 0.1 M MeCN/TBAClO$_4$ electrolyte solution at different wavelengths with the switching time of 5 s.

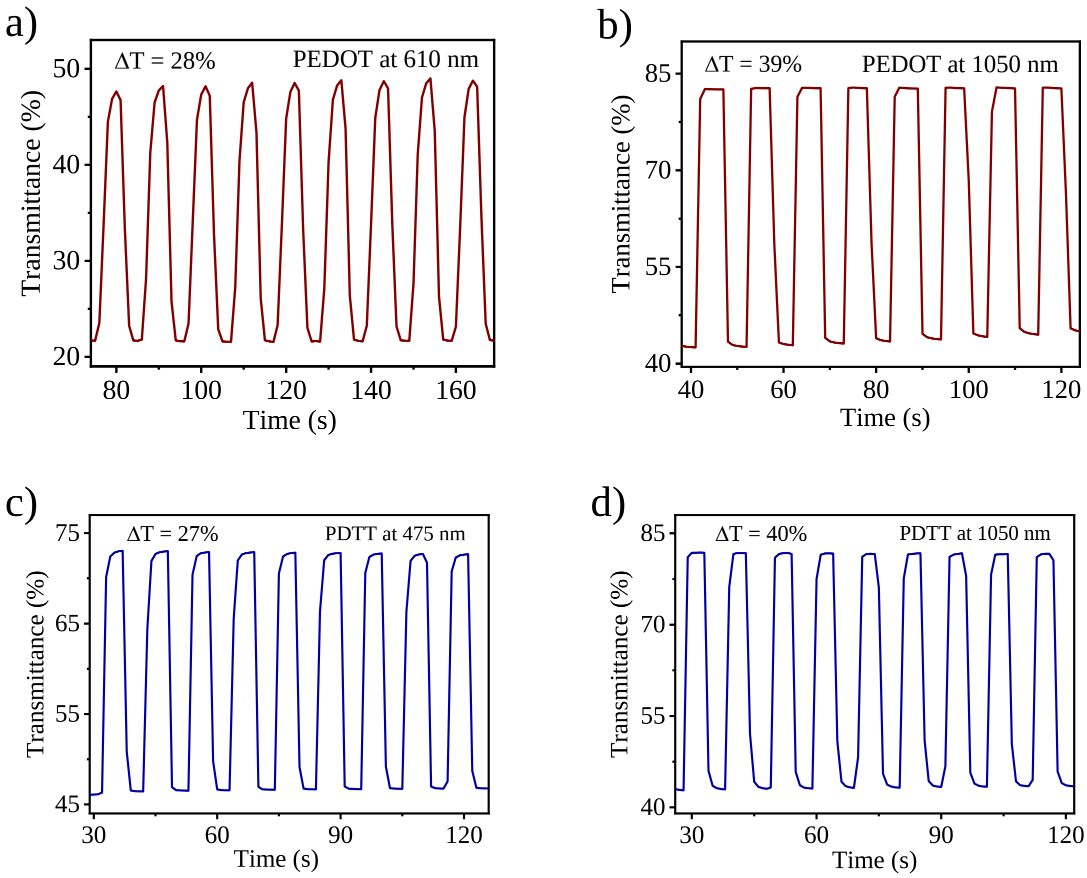

Figure S6. Optical response of the homopolymers a-b) PEDOT and c-d) PDTT in the potential between -1.0 and 1.0 V at λ_{max} and 1050 nm at the switching time of 5 s.
